# Detecting Potential Medication Selection Errors During Outpatient Pharmacy Processing of Electronic Prescriptions With the RxNorm Application Programming Interface: Retrospective Observational Cohort Study

**DOI:** 10.2196/16073

**Published:** 2020-03-11

**Authors:** Corey A Lester, Liyun Tu, Yuting Ding, Allen J Flynn

**Affiliations:** 1 Department of Clinical Pharmacy College of Pharmacy University of Michigan Ann Arbor, MI United States; 2 Department of Electrical Engineering and Computer Science College of Engineering University of Michigan Ann Arbor, MI United States; 3 Department of Learning Health Sciences School of Medicine University of Michigan Ann Arbor, MI United States

**Keywords:** patient safety, RxNorm, electronic prescription, pharmacy, pharmacists, automation

## Abstract

**Background:**

Medication errors are pervasive. Electronic prescriptions (e-prescriptions) convey secure and computer-readable prescriptions from clinics to outpatient pharmacies for dispensing. Once received, pharmacy staff perform a transcription task to select the medications needed to process e-prescriptions within their dispensing software. Later, pharmacists manually double-check medications selected to fulfill e-prescriptions before dispensing to the patient. Although pharmacist double-checks are mostly effective for catching medication selection mistakes, the cognitive process of medication selection in the computer is still prone to error because of heavy workload, inattention, and fatigue. Leveraging health information technology to identify and recover from medication selection errors can improve patient safety.

**Objective:**

This study aimed to determine the performance of an automated double-check of pharmacy prescription records to identify potential medication selection errors made in outpatient pharmacies with the RxNorm application programming interface (API).

**Methods:**

We conducted a retrospective observational analysis of 537,710 pairs of e-prescription and dispensing records from a mail-order pharmacy for the period January 2017 to October 2018. National Drug Codes (NDCs) for each pair were obtained from the National Library of Medicine’s (NLM’s) RxNorm API. The API returned RxNorm concept unique identifier (RxCUI) semantic clinical drug (SCD) identifiers associated with every NDC. The SCD identifiers returned for the e-prescription NDC were matched against the corresponding SCD identifiers from the pharmacy dispensing record NDC. An error matrix was created based on the hand-labeling of mismatched SCD pairs. Performance metrics were calculated for the e-prescription-to-dispensing record matching algorithm for both total pairs and unique pairs of NDCs in these data.

**Results:**

We analyzed 527,881 e-prescription and pharmacy dispensing record pairs. Four clinically significant cases of mismatched RxCUI identifiers were detected (ie, three different ingredient selections and one different strength selection). A total of 546 less significant cases of mismatched RxCUIs were found. Nearly all of the NDC pairs had matching RxCUIs (28,787/28,817, 99.90%-525,270/527,009, 99.67%). The RxNorm API had a sensitivity of 1, a false-positive rate of 0.00104 to 0.00312, specificity of 0.99896 to 0.99688, precision of 0.00727 to 0.04255, and F1 score of 0.01444 to 0.08163. We found 872 pairs of records without an RxCUI.

**Conclusions:**

The NLM’s RxNorm API can perform an independent and automatic double-check of correct medication selection to verify e-prescription processing at outpatient pharmacies. RxNorm has near-comprehensive coverage of prescribed medications and can be used to recover from medication selection errors. In the future, tools such as this may be able to perform automated verification of medication selection accurately enough to free pharmacists from having to perform manual double-checks of the medications selected within pharmacy dispensing software to fulfill e-prescriptions.

## Introduction

### Background

Medical error is the third leading cause of death in the United States, and medication errors are the most common type of these errors [1]. On a global basis, medication errors cost US $42 billion annually [2]. One of the medication errors with the greatest potential for harm happens when patients receive a different medication than that prescribed [3,4]. Outpatient pharmacies can dispense an incorrect medication in several ways [5]. For one, pharmacy staff members essentially transcribe e-prescription information by using software to select the medication product for dispensing based on the prescribed medication conveyed in an electronic prescription (e-prescription) [6-8]. Pharmacy software aids in the on-screen selection of a medication product from the pharmacy’s medication inventory by linking the medication data transmitted with an e-prescription to closely related drug product options. To identify the prescribed drug product, e-prescriptions carry a representative National Drug Code (NDC) along with a standard drug description from a commercial drug compendium (eg, DailyMed). Sometimes, when one exists, a corresponding RxNorm concept unique identifier (RxCUI) is also included in transmitted e-prescriptions for drug identification.

There are instances when pharmacy staff enter e-prescription medication information manually, bypassing available product identification safety features [8]. In these cases, pharmacy staff can type the name of any medication held in inventory into a new prescription record. This increases the risk of entering an incorrect medication into the pharmacy’s software and dispensing the wrong medication to the patient. An independent double-check by a pharmacist helps identify these medication selection errors. However, the pharmacist can still miss these errors as well because of fatigue, workload, or stress [9-12]. To support pharmacists, an independent, automated double-check of the medications selected to fulfill e-prescriptions could identify medication selection errors post hoc. The electronic transmission of e-prescriptions, coupled with electronic representations of prescriptions in pharmacy dispensing systems, enables machines to perform novel safety checks not possible in the past with handwritten or verbal prescriptions.

RxNorm, the US National Library of Medicine (NLM) drug terminology system, has the potential to detect medication selection mistakes and prevent patient harm. Bell et al [13] demonstrated the comprehensiveness of RxNorm by finding an RxCUI for all but 1 of 19,743 sample e-prescriptions. They also reported an RxCUI mismatch rate of 3.4% between e-prescription and pharmacy dispensing records. Most mismatches between the medication prescribed and dispensed were deemed not of clinical significance. One limitation of previous work is their use of downloadable .csv files of RxNorm content. RxNorm content is also available through an application programming interface (API). This API is important because it can support continuous detection of medication selection errors in pharmacy practice, making it possible to identify and resolve errors before they reach the patient. To demonstrate this, we make use of the publicly available RxNorm API to perform checks of past dispensing records.

### Objective

The objective of this study was to establish an automated RxNorm API double-check and evaluate its performance as a method for detecting potential medication selection errors occurring in outpatient pharmacies. This study contributes a blueprint for how to do this with the widely available API resource from NLM.

## Methods

### Overview

We analyzed e-prescription and corresponding dispensing records that were transmitted through a mail-order pharmacy in the United States. NDCs for each medication were matched mainly to its associated semantic clinical drug (SCD) RxCUI identifiers using the NLM’s RxNorm API. Semantic branded drug (SBD) and generic pack (GPCK) RxCUIs were also used. A direct match was performed between e-prescription and pharmacy system dispensing record SCDs. Afterward, all mismatched pairs found were hand-labeled into categories. Finally, performance metrics were applied to evaluate the e-prescription-dispensing record matching algorithm for both total pairs and unique pairs of NDCs in the data. We use a prototypical example with a specific NDC pair in this section to communicate how the system functions (ie, NDC=00093-5117-98; Diltiazem HCl ER coated beads 180 mg and NDC=00008-0841-81; Protonix 40 mg oral tablet).

### Data Source

We obtained 537,710 pairs of e-prescription and pharmacy dispensing records over the period of January 2017 to October 2018. For e-prescriptions, the variables included the free-text medication name, an alpha-numeric NDC (ie, these are representative NDCs required to transmit an e-prescription), and national provider identifier (NPI). A corresponding dispensing record for data appearing on the prescription label included the free-text medication name and an alpha-numeric NDC (ie, the specific product dispensed to the patient). To determine the origin of the e-prescription, we linked our prescriber NPI to the Centers for Medicare and Medicaid’s National Plan and Provider Enumeration System NPI download file (November 2018 release) [14]. We used no personally identifiable patient-specific data. The University of Michigan Institutional Review Board reviewed our study protocol and assigned exemption status.

### Data Filtering and Cleaning

**Figure 1:**
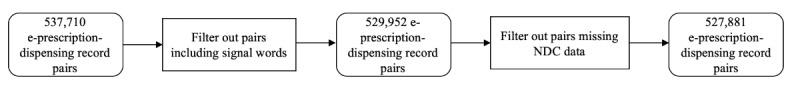
Data cleaning process. e-prescription: electronic prescription; NDC: National Drug Code.

To ensure we included only valid e-prescription-dispensing record pairs, we performed a series of data filtering and cleaning tasks. We removed e-prescription-dispensing record pairs containing certain signal words: *duplicate, cancel, wrong, and denied* from the data. We also removed pairs containing missing NDC data. When necessary, we padded NDC codes with leading zeros to make them conform to a standard 11-digit format. This step normalized NDC codes and prepared them for the NLM’s RxNorm API. Figure 1 shows the results of our data cleaning steps.

### Data Analysis

We conducted a three-step analysis. The three steps of the analysis were (1) linking NDCs to SCD RxCUIs via the RxNorm API, (2) matching SCD RxCUIs in each e-prescription-dispensing record pair, and (3) calculating performance metrics. Besides reporting the results of the three-step analysis, we also report the most common medications dispensed by the mail-order pharmacy as well as the most common medication classes represented in our dataset. As the dataset came from a mail-order pharmacy, the dataset does not include injectable products exclusively used in inpatient or long-term care settings.

**Figure 2:**
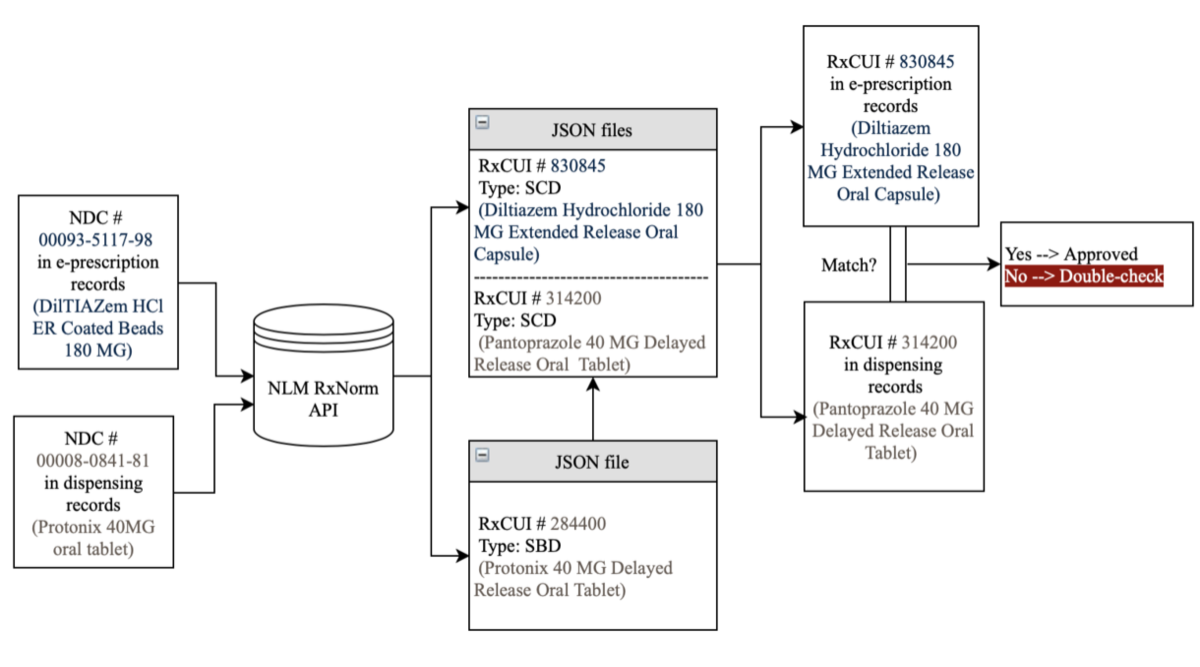
Proposed system for detecting medication selection errors of e-prescriptions with RxNorm application programming interface. A prototypical example is included. API: application programming interface; e-prescription: electronic prescription; JSON: JavaScript object notation; NDC: National Drug Code; NLM: National Library of Medicine; RXCUI: RxNorm concept unique identifier; SBD: semantic branded drug; SCD: semantic clinical drug.

Moving from left to right in Figure 2, the first step in our analysis mapped the NDC conveyed by the e-prescription and the NDC on the pharmacy’s dispensing record to a corresponding SCD (or SBD or GPCK or branded pack) RxCUI. The automated checking system we created called the RxNorm API for “ndcstatus” to do this mapping [15]. When called in this manner using an NDC number, the RxNorm API returns a corresponding RxCUI for all current and retired NDC numbers passed to the API. According to the NLM RxNorm Technical Documentation, there is a one-to-many relationship between RxCUIs and NDC numbers [16]. Thus, no ambiguity should exist in this mapping process.

Figure 2 shows an overview of the analytic process and proposed future automated checking system. The figure also includes an embedded example of e-prescription record that failed to match its corresponding dispensing record.

We performed additional identification of RxCUI term types to ensure RxCUIs in e-prescription and dispensing records were comparable. As SCD RxCUIs uniquely identify instances of drug name, strength, and form for the majority of drug products with NDCs, we chose to perform the comparison between corresponding SCD terms. If a term type other than SCD (eg, an SBD or GPCK) was returned by the RxNorm API (API method *rxcui/{rxcui}/properties*), we would call the API a second time (API method *rxcui/{rxcui}/related?tty=SCD*) to identify all SCDs corresponding to the SBD or GPCK in those cases [15]. For example, in Figure 2, the NDC 00008-0841-81 found in a dispensing record is mapped to SBD RxCUI 284400, which is linked using the RxNorm API to SCD RxCUI 314200.

The second step of analysis used a matching algorithm to compare RxCUI returned for each e-prescription and dispensing record pair. The algorithm identified pairs with matched and unmatched RxCUIs. The output divided pairs into three categories: pairs with missing RxCUIs, pairs with matching RxCUIs, and pairs with nonmatching RxCUIs.

We report examples of actual NDC numbers for those pairs missing a corresponding RxCUI from RxNorm. It is important to note that manufacturers can generate new NDCs on their own. The majority, but not all NDCs, are curated by NLM and appear in RxNorm.

For the other two categories of pairs of e-prescription and dispensing records, we analyzed incorrect medication selection using an *error matrix*. We labeled the outcomes of our matching algorithm in the following way:

- True positives (TPs): SCD mismatch with incorrect drug/strength
- False negatives: Nonequivalent drugs with matching SCD
- False positives (FPs): SCD mismatch with incorrect quantity/form/qualitative distinction/releasing mechanism or outside RxNorm
- True negatives: Equivalent drugs with matching SCD

In our running example that is also embedded in Figure 2, a mismatch between SCD RxCUI 830845 for the drug diltiazem and SCD RxCUI 314200 for the drug pantoprazole is found. Diltiazem and pantoprazole are different medications and so a medication selection error has been detected by the system. Therefore, in our analysis, this example would be classified as a TP in the scheme above.

As a final step, to evaluate the automated checking system’s overall performance, we analyzed unique pairs of NDCs and total NDC pairs. This allowed us to consider how the system might learn over time to reduce nuisance alerts in the pharmacy. For each specific mismatched e-prescription-dispensing record pair, we classified the mismatched pair as clinically significant or not. In a learning automated checking system, once a mismatch is tagged as nonclinically significant, the system should ignore that mismatch in the future and not fire an alert to pharmacists on subsequent mismatches of that pair [17].

For each unique NDC pair with mismatched RxCUIs, we identified the type and frequency of the mismatch. These types of mismatches included incorrect ingredient, incorrect strength, incorrect quantity, incorrect dosage form (eg, capsule vs cream), and problematic RxCUIs. We defined a true positive as a different ingredient selection or a different strength selection (eg, 25 mg vs 50 mg). These errors signaled a TP error because the different ingredient or strength could lead to patient harm [18,19]. We defined an FP as an incorrect medication selection that would typically not lead to patient harm (ie, different quantity, different dosage form, or problematic RxCUI). Using the error matrix, we calculated the following performance metrics: accuracy, sensitivity/recall, false-positive rate, specificity, precision, and F1 score.

We report these performance metrics for both the unique pairs of NDCs and for the total pairs of NDCs in the dataset. This allows us to consider the automated checking system’s performance if we deactivated alerts for specific mismatched RxCUI pairs after a pharmacist overrides them once (ie, as if the system operated as a learning system). For total pairs, we report performance of the automated checking system if the same mismatched RxCUI pair creates an alert every time the pharmacy dispensed the mismatched RxCUI pair (ie, as if the system operated as a static system that could not learn).

## Results

The processed dataset included 527,881 (527,881/537,710, 98.17%) of the original dataset) e-prescription-dispensing record pairs from 64,805 prescribers in all 50 US states. There were 17,123 unique NDCs and 3,838 unique SCDs in the prescription-dispensing record pair data. The most frequently dispensed medications were atorvastatin, amlodipine, hydrochlorothiazide, and omeprazole (Table 1). The most common therapeutic drug classes in the dataset were cholesterol-lowering agents, which accounted for 118,877 pairs (118,877/527,881, 22.52%). The next most commonly analyzed therapeutic drug classes were blood glucose–lowering agents for 46,553 pairs (46,553/527,881, 8.82%) and antidepressants for 44,586 pairs (44,586/527,881, 8.45%).

**Table 1:** Five most frequently found clinical drug packs in the analyzed dataset (N=527,881).

RxCUI	Semantic clinical drug name	Matched pairs, n (%)
617311	Atorvastatin 40 mg oral tablet	7688 (1.46)
617310	Atorvastatin 20 mg oral tablet	7683 (1.46)
198051	Omeprazole 20 mg delayed release oral capsule	7674 (1.45)
197361	Amlodipine 5 mg oral tablet	6631 (1.26)
310798	Hydrochlorothiazide 25 mg oral tablet	6252 (1.18)

The first step of our analysis mapped each pair of NDC codes arising from an e-prescription-dispensing record pair to their related SCD RxCUI. We found 872 pairs with 1731 NDCs where the RxNorm API did not return an RxCUI. Table 2 contains a list of examples with NDC codes that did not map to an RxCUI. One NDC code sometimes corresponded to more than one medication product description within the e-prescription data. Examples of medications with these unmatched NDC numbers are *multivitamin tablet*, *blood sugar diagnostic strip*, and *Lancets Miscellaneous*. We excluded these 872 pairs from further analysis steps.

**Table 2:** Ten most frequent National Drug Codes without a corresponding RxNorm concept unique identifier (n=1731).

National Drug Code	Frequency, n (%)	Sample medication name^a^
0000-20002-02	440 (25.42)	Cyanocobalamin (Vitamin B-12) 100 mcg tablet
0000-00000-07	344 (1987)	Blood glucose test strips
0000-00000-08	220 (12.71)	Lancets 28 gauge
0000-00000-09	108 (6.24)	Blood glucose meter
2743-40010-21	38 (2.20)	Magnesium oxide 400 mg capsule
9289-60000-08	36 (2.08)	Blood glucose meter kit
0888-16096-00	34 (1.96)	Insulin syringe-needle U-100 1 ml 30 gauge × 5/16 syringe
3841-50003-08	34 (1.96)	Insulin pen needle 30 g × 8 mm
9403-00002-02	30 (1.73)	Embrace blood glucose system strips
0862-70014-01	22 (1.27)	Dexcom G5 mobile transmitter

^a^Some National Drug Codes correspond to multiple medication product descriptions.

As a result of the matching process performed by the automated checking system, 0.10% (550/527,009) pairs had different SCDs for their e-prescription and corresponding dispensing records. Table 3 reports on these 550 mismatched pairs by issue category. Three mismatched pairs showed different ingredients. One mismatched pair contained different strengths of the correct medication. Of the other 546 mismatched pairs found, 347 had concept names different in one or more essential term, including quantity (ie, number of units in a pack) or form (eg, solution for injection vs prefilled syringe). The other 199 were either special cases (eg, sugar-free vs not sugar-free) or were cases with NDCs that were not curated by RxNorm, meaning that the NDCs appeared in RxNorm but came from a vocabulary source other than DailyMed or First Data Bank [16,20].

**Table 3:** Performance evaluation of the algorithm for detecting different medication selection, with examples (n=527,009).

Issues	Frequency, n (%)	Electronic Rx (E-Rx) RxNorm concept unique identifier (RxCUI)	Medication conveyed by E-Rx	Pharmacy prescription label RxCUI	Medication dispensed by pharmacy
Same medication	526,457 (99.90)	751620	Bystolic 2.5 mg oral tablet	751620	Bystolic 2.5 mg oral tablet
Different ingredient	3 (0.00)	313585	24 HR venlafaxine 75 mg extended release oral capsule	966225	Levothyroxine sodium 0.15 mg oral tablet
Different strength	1 (0.00)	861700	Amylases 82000 UNT/endopeptidases 51000 UNT/lipase 15000 UNT delayed release oral capsule	1595476	Amylases 84000 UNT/endopeptidases 63000 UNT/lipase 20000 UNT delayed release oral capsule
Different pack quantity	285 (0.05)	905100	12 (risedronate sodium 35 mg oral tablet) pack	905092	4 (risedronate sodium 35 mg oral tablet) pack
Different form	67 (0.01)	835840	testosterone cypionate 200 mg/mL injectable solution	2047882	1 mL testosterone cypionate 200 mg/mL injection
Concept outside RxNorm	40 (0.01)	1371671	Proprietary	1371861	Proprietary
Others (eg, qualitative distinctions)	159 (0.03)	198034	24 HR nifedipine 30 mg extended release oral tablet	1812011	Osmotic 24 HR nifedipine 30 mg extended release oral tablet

**Figure 3:**
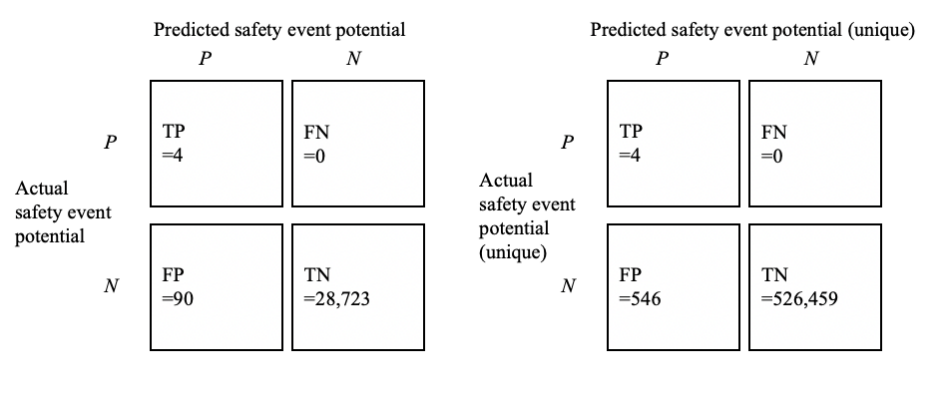
Results of medication safety event error matrix with and without duplicate e-prescription-dispensing record pairs.

After removing duplicates, 0.33% (94/28,817) unique e-prescription-dispensing record pairs contained a mismatched RxCUI. Figure 3 shows the error matrix for clinically significant incorrect medication selection detection from the data. Table 4 reports performance with and without duplicate mismatched e-prescription-dispensing record pairs.

**Table 4:** Algorithm performance with and without duplicate electronic prescription–dispensing record pairs.

Metrics	Unique electronic prescription (e-prescription)–dispensing record pairs (n=28,817)	Total e-prescription-dispensing record pairs (n=527,009)^a^
Accuracy	0.99688	0.99896
Sensitivity/recall	1.00000	1.00000
False-positive rate	0.00312	0.00104
Specificity	0.99688	0.99896
Precision	0.04255	0.00727
F1 Score	0.08163	0.01444

^a^Pairs including duplicate dispensing of the same National Drug Code pair.

## Discussion

### Principal Findings

This study demonstrates a straightforward method of using the publicly available US NLM RxNorm API to identify potential medication selection errors made during transcription and processing of e-prescriptions in outpatient pharmacies. We evaluated the performance of an automated comparison for e-prescription-dispensing record pairs of medications ordered and dispensed using a dataset from a mail-order pharmacy. The automated checking system we developed identified 550 cases (550/527,009, 0.10%) of mismatched e-prescription-dispensing record pairs with issues ranging from incorrect medications to problems with unresolved NDCs. This rate is low when compared with a study reporting a 3.4% mismatch rate using RxNorm [13]. One potential reason for this is that the pharmacies sampled in the previous study were largely community pharmacies and may have different dispensing software, physical environments, and workloads.

### Clinical Implications

To promote correct dispensing of medication, the RxNorm SCD is recommended by the National Council for Prescription Drug Programs (NCPDP) SCRIPT standards (currently 10.6), when one exists, to be included with the transmission of all e-prescriptions [21]. Unlike NDCs, RxNorm codes are centrally managed, making them easier to resolve to an actual drug product and potentially more suitable as a drug product identifier data standard. Including SCDs with all e-prescriptions can help to ensure that the meaning of the clinical drug product information is communicated correctly to the pharmacy so that pharmacy staff can select the drug product that they will dispense to the patient accurately. Besides, as more health information technology applications consume and process e-prescription information, having SCDs included in e-prescriptions will help protect the public from unintended software errors.

A 2014 national sample of e-prescriptions revealed that only 33.0% (n=49,997) were transmitted with an SCD in the HL7 message, even though the e-prescribing network allows for transmitting these identifiers. Since then, the adoption of SCD RxCUIs has increased significantly as a result of Office of National Coordinator supporting the NCPDP SCRIPT standards [22] and more recently, due to the Centers for Medicare and Medicaid Services requiring the use of NCPDP SCRIPT standards for conveying e-prescriptions [21]. The use of a representative NDC to convey a prescribed drug product via an e-prescription is too specific, and NDCs are also potentially stale and no longer accurate [23]. In the pharmacy, staff are responsible for selecting the exact NDC of the drug product that is dispensed to the patient. Inclusion of the RxNorm SCD can aid the transcription of e-prescription information into pharmacy systems, resulting in fewer dispensing errors. As an added benefit, our automated checking system can be extended to the international community based on the recent expansion of RxNorm to integrate with other medication terminology standards, such as the Identification of Medicinal Products used by the European Medicines Agency and DrugBank used in Canada [24].

Consistent with previous literature, the evident rate of errors that reached the patient because of incorrect medication selection was approximately 0.1% [25]. We detected different ingredient errors, different medication strength errors, dosage form errors, and errors in the quantity to be dispensed. These are all common types of dispensing errors reported by community pharmacies around the world [26-31]. These errors differ in their potential to cause harm. In the worst case, when the wrong drug is dispensed, two kinds of harm may follow. First, in such a case, an individual does not benefit from getting the correct drug until the error is discovered. Second, the individual is exposed to a different drug. In other cases, for example, when the wrong package size of the correct drug is dispensed, the harm that may result is less, although such an error can lead to confusion for the patient. Owing to these important differences in the potential of errors to cause harm, in the future, we plan to explore methods of quantifying harm averted as well as errors avoided.

This study demonstrates the potential for the RxNorm API to enable an automated double-check of e-prescription and dispensing record information that could substitute for many of the manual double-checks provided by pharmacists today. Instead of having pharmacists double-check the fidelity of every e-prescription entered into the pharmacy system, the number requiring a manual double-check might be reduced 1000-fold or more, saving pharmacists’ worktime and cognitive effort [9]. In addition, the efficiency and reliability of the RxNorm-enabled matching algorithm suggest it may be feasible to apply this type of automated checking at different stages in the medication dispensing workflow. For example, using the RxNorm API in real time to fire an alert if the technician makes an incorrect drug selection. Alternatively, automated checking could trigger alerts to an off-site staff member to determine the clinical significance of the problem before interrupting the workflow in the pharmacy.

However, there is a potential for alert fatigue by using this type of automated checking. A previous study found that pharmacy staff were annoyed by false-positive alerts but also that their satisfaction with alerting goes up when given opportunities to prevent dispensing errors [13]. We found that just 4 out of the 550 cases of mismatched pairs were of clinical significance in this study. This means there were 137 false alerts for every clinically significant one. To overcome alert fatigue, we are interested in developing a learning capability to complement the automated checking capability demonstrated here. If the current automated checking system could learn the difference between mismatched pairs that are clinically significant and those that are not, then we could suppress alerts for insignificant mismatches. Our tests show that in this case, given the sequence of prescribing events, there is the potential to lower the number of alerts needed to surface a clinically significant issue from 138 (137:1) to 24 (23:1). Over time, continuing to learn from the mismatched pairs identified would further lower the ratio of false-positive to true-positive alerts. We believe this type of learning approach could decrease the chance of alert fatigue [32].

Previous studies have demonstrated a variety of uses for the RxNorm drug terminology system, including improving medication history taking, resolving free-text medication naming conventions from electronic health records (EHRs), and matching clinical drug names across medication formularies [33-35]. RxNorm provides a distinct advantage over using NDCs in that the RxNorm’s RxCUIs provide normalization of drug naming concepts across systems with different configurations. This benefit from RxNorm is particularly relevant for EHRs and pharmacy dispensing software. EHRs are configured with different medication naming conventions. When different naming conventions are associated with the same NDCs, ambiguity arises in the e-prescription data ecosystem upon which pharmacies depend. RxNorm provides a means to reconcile the different ways that medication information is configured in EHRs, helping to address and remove unwanted ambiguity from the e-prescription data ecosystem. Using RxNorm RxCUIs with e-prescriptions and dispensing records improves the quality of data and helps ensure accurate transmission of e-prescription information.

### Future Work

To validate our findings, a logical next step is to expand the use of automated checking to process a larger dataset drawn from multiple pharmacies. Given that 1.91 billion new e-prescriptions are transmitted annually in the United States, other future work needs to establish a reliable technical infrastructure for outpatient pharmacies to analyze e-prescription-dispensing record pairs routinely with the existing capabilities of the RxNorm API. One solution to prevent the worst medication selection errors would be to use the RxNorm API to link an NDC from the e-prescription to a list of NDCs mapped to the same SCD in the pharmacy dispensing software. Once these capabilities are further demonstrated and made widely available, additional research should focus on other areas where automated checking can have this kind of positive impact.

### Limitations

These findings are limited primarily because the dataset came from a single mail-order pharmacy in the United States. Although the e-prescriptions received by this pharmacy came from all over the country, the dataset is not a representative national sample of e-prescription and dispensing records. These data also only contain medications used in the outpatient setting (ie, not injectable or infusion products using in inpatient or long-term care settings). A second limitation is that although our algorithm detected seeming clinically significant medication safety incidents, it is possible that there were other plausible explanations for these mismatches (eg, the case of a pharmacist who receives verbal approval from a prescriber to change the medication product). Another concern is that drug concepts and their codes are only valid for a limited period of time. During our analysis, we have checked and remapped obsolete NDCs and RxCUIs to active concept unique identifiers. But the reuse of an NDC number can lead to problems when combining datasets. Today, the Food and Drug Administration (FDA) allows firms to reassign an NDC 5 years after the expiration date of a discontinued drug [36]. As the FDA does not restrict the creation of NDCs, some codes fall outside the intended scope of RxNorm (eg, proprietary prescription compounds or diabetic testing supplies). This means that some NDCs will never be analyzable with the proposed RxCUI matching system.

### Conclusions

In this study, we used the NLM’s RxNorm API to enable accurate automated checks of e-prescription-dispensing record pairs. We identified a small but critical number of e-prescription processing errors made at the pharmacy. Our method can detect potential dispensing errors before they cause harm and, if combined with other machine-checking interventions, has the potential to eliminate the need for the majority of the manual pharmacist double-check to compare the e-prescription medication product with the medication product selected for dispensing. This type of automation can help reduce the risk of dispensing the incorrect medication to the patient while sparing the pharmacist’s worktime for higher value patient care tasks. Using these results, validation in other pharmacies is necessary before the widespread adoption of this system as one part of a safety management system that relies on the retrospective analysis of e-prescription and dispensing record data.

## Abbreviations

API: application programming interface
EHR: electronic health record
e-prescription: electronic prescription
FDA: Food and Drug Administration
FP: false positive
GPCK: generic pack
NCPDP: National Council for Prescription Drug Programs
NDC: National Drug Code
NLM: National Library of Medicine
NPI: National Provider Identifier
RxCUI: RxNorm concept unique identifier
SBD: semantic branded drug
SCD: semantic clinical drug
TP: true positive
